# GITRL modulates the activities of p38 MAPK and STAT3 to promote Th17 cell differentiation in autoimmune arthritis

**DOI:** 10.18632/oncotarget.6535

**Published:** 2015-12-09

**Authors:** Xinyi Tang, Jie Tian, Jie Ma, Jiemin Wang, Chen Qi, Ke Rui, Yungang Wang, Huaxi Xu, Liwei Lu, Shengjun Wang

**Affiliations:** ^1^ Department of Laboratory Medicine, The Affiliated People's Hospital, Jiangsu University, Zhenjiang, China; ^2^ Institute of Laboratory Medicine, Jiangsu Key Laboratory of Laboratory Medicine, Jiangsu University, Zhenjiang, China; ^3^ Department of Pathology, The University of Hong Kong, Hong Kong, China

**Keywords:** GITRL, p38 MAPK, STAT3, Th17 cells, autoimmune arthritis, Immunology and Microbiology Section, Immune response, Immunity

## Abstract

The glucocorticoid-induced TNFR family-related protein (GITR) and its ligand play a critical role in the pathogenesis of autoimmune arthritis by enhancing the Th17 cell response, but their molecular mechanisms remain largely unclear. This study aims to define the role of p38 mitogen-activated protein kinases (MAPK) and signal transducer and activator of transcription 3 (STAT3) signaling in GITRL-induced Th17 cells in autoimmune arthritis. We found that the p38 phosphorylation was enhanced by GITRL in activated CD4^+^T cells, and the p38 inhibitor restrained the GITRL-induced Th17 cell expansion in a dose-dependent manner. Moreover, there was decreased STAT3 activity on Tyr705 and Ser727 with the p38 inhibitor in vitro. Notably, the p38 inhibitor could prevent GITRL-treated arthritis progression and markedly decrease the Th17 cell percentages. The phosphorylation of the Tyr705 site was significantly lower in the GITRL-treated CIA mice administrated with the p38 inhibitor. A significantly higher phosphorylation of p38 was detected in RA patients and had a positive relationship with the serum level of anti-cyclic citrullinated peptide (anti-CCP) antibody. Our findings have indicated that GITRL could promote Th17 cell differentiation by p38 MAPK and STAT3 signaling in autoimmune arthritis.

## INTRODUCTION

Rheumatoid arthritis (RA) is an autoimmune disease characterized by chronic inflammation of synovial joints and the progressive destruction of cartilage and bone tissue, ultimately leading to disability [1]. Th17 cells, a subset of helper T cell populations that selectively produce IL-17, have developed a reputation as a critical element in RA patients [2, 3] and collagen-induced arthritis (CIA) mice [4]. Accumulated data have shown that TGF-β with IL-6 and IL-23 instruct Th17 cell differentiation *via* RORγt and STAT3 [5, 6], and IL-21 is needed for the expansion of Th17 cells in autocrine signaling [7]. Furthermore, STAT3 is a vital transcription factor for Th17 cell differentiation by directly binding and regulating Il17a and the Il21 locus, as well as regulating RORγt expression [8, 9].

The murine glucocorticoid-induced tumor necrosis factor receptor-related protein (GITR) had been described in 1997 as a dexamethasone-inducible molecule in T cells [10]. A low level of GITR is constitutively expressed on effector T cells and increases upon activation [11]. However, regulatory T cells (Treg) constitutively express high levels of GITR, and GITR ligand (GITRL) can abrogate the suppressive function [12, 13]. GITRL is expressed on antigen-presenting cells (APCs), such as DCs, macrophages, and B cells [14, 15]. A recent study demonstrated a marked expansion of Th17 cells when induced from naïve CD4^+^T cells cultured with GITRL protein. Moreover, an administration of recombinant GITRL in CIA mice enhanced Th17 cell generation and exacerbated arthritis development [16]. However, the molecular mechanisms underlying GITRL modulation of Th17 cells remain largely unclear. Current studies have shown GITR cross-linking provided costimulation of naïve and activated T cells and resulted in activation of MAPKs[17, 18]. P38 MAPK is a member of MAPK family, and activation of p38 MAPK signaling in CD4^+^T cells plays a pivotal role in Th17 cell function by regulating IL-17 production [19-21].

In this study, we firstly found that the cross-linking of GITR triggered by GITRL provided an enhanced phosphorylation of p38 MAPK and further induced the phosphorylation of STAT3 in activated CD4^+^T cells. We also demonstrated that Th17 cell differentiation induced by GITRL protein could be suppressed after culturing Th17 cells with a p38 MAPK inhibitor. Moreover, the promotion of arthritis by mGITRL in collagen-immunized mice could be relieved by administering a p38 MAPK inhibitor. Furthermore, elevated levels of p38 MAPK phosphorylation were detected in CD4^+^T cells from the peripheral blood of RA patients, which displayed a significant correlation with increased serum levels of anti-CCP antibody in these patients. Thus, these results have revealed an important pathway for Th17 cell differentiation induced by GITRL and a previously unappreciated role of p38 MAPK in the pathogenesis of autoimmune arthritis.

## RESULTS

### P38 MAPK is necessary for GITRL-induced Th17 differentiation

**Figure 1:**
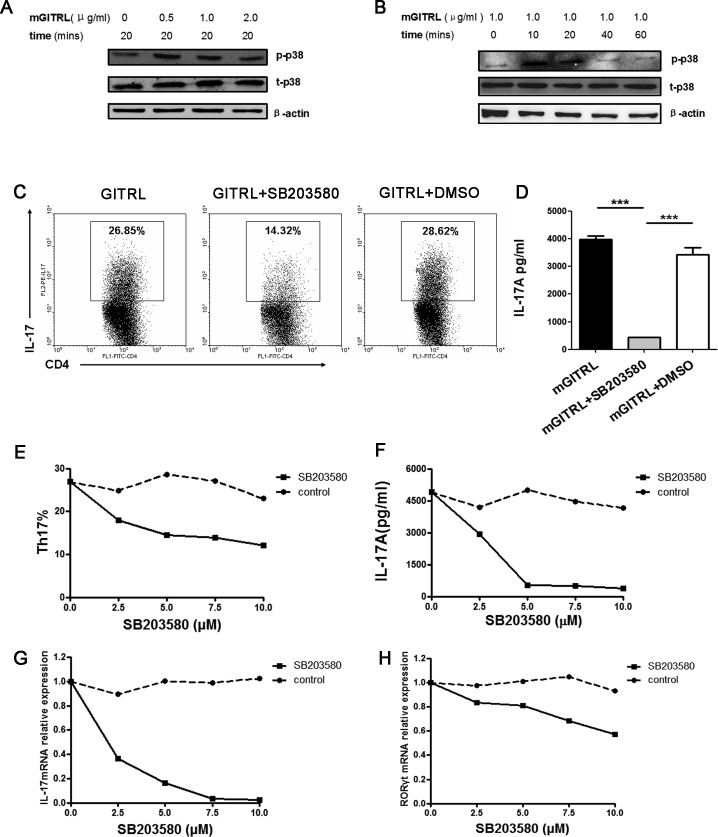
p38 MAPK is necessary for GITRL-induced Th17 differentiation **A.** Naïve CD4^+^T cells were activated by an anti-CD3 mAb (1 μg/mL) and GITRL protein (1 μg/mL) for 72 hours. The activated cells were washed and restimulated with different concentrations of GITRL protein (0, 0.5, 1.0, 2.0 μg/mL) at 37°C for 20 min. The phosphorylation of p38 MAPK was detected by Western blot. **B.** Naïve CD4^+^T cells were activated by anti-CD3 mAb (1 μg/mL) and GITRL protein (1 μg/mL) for 72 hours. The activated cells were washed and restimulated with GITRL protein (1 μg/mL) at 37°C for 10-60 min. The phosphorylation of p38 MAPK was detected by Western blot. **C., D.** Naïve CD4^+^T cells were cultured with TGF-β (2.5 ng/mL), IL-6 (30 ng/mL), IL-23 (30 ng/mL), anti-IFN-γ (5 μg/mL), anti-IL-4 (5 μg/mL) and GITRL protein (1 μg/mL) in a 24-well plate precoated with anti-CD3 mAb (1 μg/mL) for 72 hours in the presence or absence of the p38 MAPK inhibitor SB203580 (5 μM); the DMSO was the solvent of SB203580. The frequencies of Th17 cells in cultures with different treatments were analyzed by FCM **C.** The concentration of IL-17 in the culture supernatant was detected by ELISA **D., E., F., G., H.** Naïve CD4^+^T cells were cultured under Th17 cell differentiation conditions and treated with GITRL protein (1 μg/mL) for 72 hours in the presence of SB203580 (0, 2.5, 5, 10 μM) or the solvent control. The frequencies of Th17 cells in cultures with different treatments were analyzed by FCM **E.**. The concentrations of IL-17 in the culture supernatant was detected by ELISA **F.**; relative expression of IL-17 **G.** and RORγt **H.** mRNA were detected by qRT-PCR. Data are mean±SD. ***, *P* < 0.001.

To characterize p38 MAPK signaling pathways that may contribute to GITRL-induced cellular effects, we analyzed the phosphorylation of p38 MAPK in activated T cells using different concentrations of GITRL protein. When stimulated with 0.5 or 1.0 μg/ml GITRL protein, the activated CD4^+^T cells had higher phosphorylation of p38 MAPK (Figure 1A). After that, we analyzed the phosphorylation of p38 MAPK in CD4^+^T cells using GITRL protein (1.0 μg/ml) for 10, 20, 40, and 60 min. The results show that the phosphorylation of p38 was enhanced when stimulated with 1.0 μg/ml GITRL protein for 10 or 20 min (Figure 1B).

Next, we investigated if p38 MAPK had an effect on GITRL-induced Th17 cell differentiation. Naïve CD4^+^T cells were induced with anti-CD3 mAb and GITRL protein under Th17 differentiation conditions in the presence of varying concentrations of the p38 MAPK inhibitor (0, 2.5, 5, 7.5, and 10 μM). There was a clear decrease of the proportion of Th17 cells in the presence of the p38 MAPK inhibitor (Figure 1C). Compared with the supernatant from developing Th17 cells induced by GITRL, IL-17 concentration was significantly reduced in the presence of p38 MAPK inhibitor (Figure 1D). Additionally, Th17 cells frequency, IL-17 concentration and associated factors of mRNA expression were decreased in a dose-dependent manner by the p38 MAPK inhibitor during the GITRL-induced Th17 cell differentiation (Figure 1E-1H). These results indicated that p38 MAPK is necessary for GITRL-induced Th17 cell differentiation.

### GITRL promotes the phosphorylation of STAT3 on Tyr705 and Ser727 *via* p38 MAPK in Th17 cell differentiation

The classical JAK-STAT3 signaling pathway and Tyr705 phosphorylation are characteristic of STAT3 activation and a requisite condition of Th17 cell differentiation. Apart from the Tyr705 site, Ser727 is another important phosphorylation site of STAT3 [23-25]. Previous studies have found p38 MAPK could promote the phosphorylation of STAT3 on Ser727 [26, 27].

**Figure 2:**
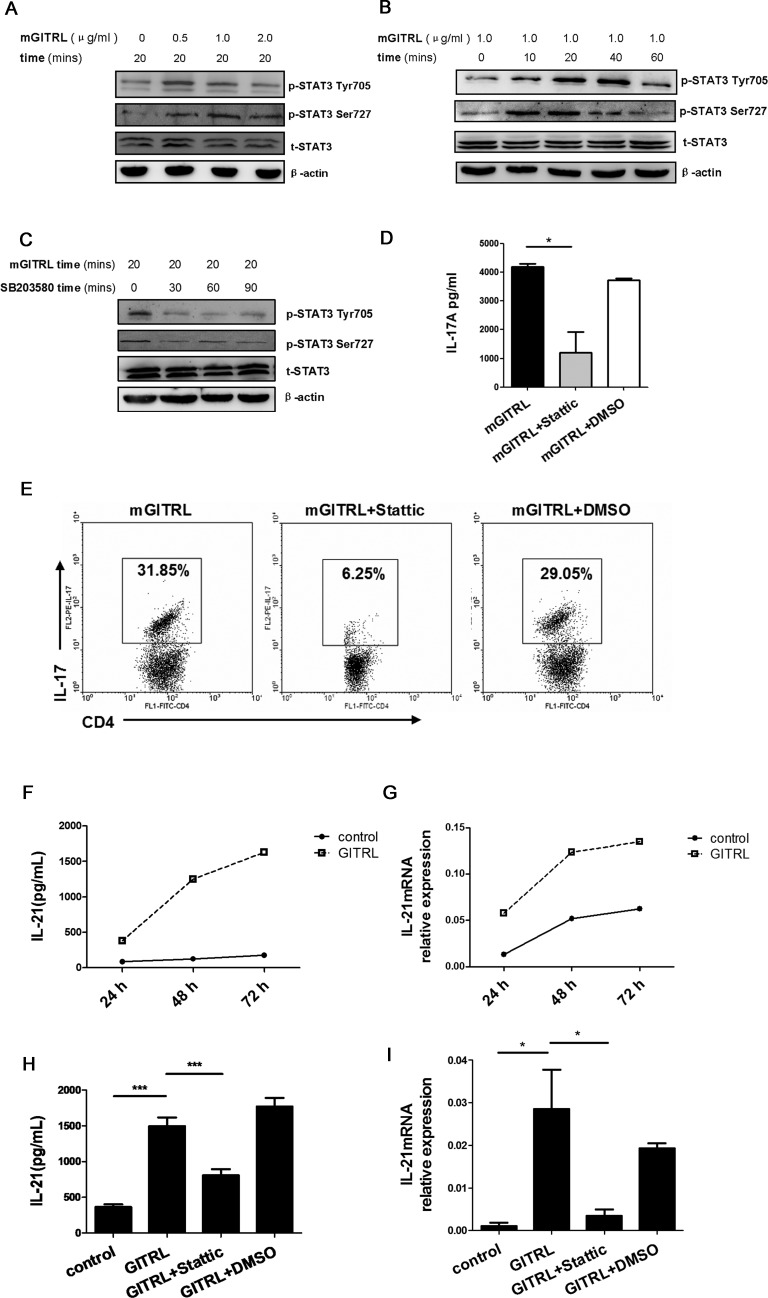
GITRL protein promotes the phosphorylation of STAT3 on Tyr705 and Ser727 *via* p38 MAPK in Th17 cell differentiation **A.** Purified Naïve CD4^+^T cells were activated by anti-CD3 mAb (1 μg/mL) and GITRL protein (1 μg/mL) for 72 hours. The activated cells were washed and restimulated with different concentrations of GITRL protein at 37°C for 20 min. The phosphorylation of STAT3 Tyr705 and Ser727 was detected by Western blot.**B.** The activated CD4^+^T cells were restimulated with GITRL protein (1 μg/mL) at 37°C for different lengths of time. The phosphorylation of STAT3 Tyr705 and Ser727 was detected by Western blot.**C.** The activated CD4^+^T cells were pretreated with SB203580 (5 μM) for 30, 60, 90, and 120 min and restimulated with GITRL protein (1 μg/mL) for 20 min at 37°C. The phosphorylation of STAT3 Tyr705 and Ser727 was detected by Western blot.**D., E.** Naïve CD4^+^T cells were cultured under Th17 condition as previously described and treated with GITRL protein (1 μg/mL) for 72 hours in the presence or absence of the STAT3 inhibitor Stattic (20 μM). DMSO was used as the solvent of Stattic. The concentration of IL-17 in the culture supernatant was detected by ELISA **D.**. The frequencies of Th17 cells in the cultures with the different treatments were analyzed by FCM **E., F., G.** Naïve CD4^+^ T cells were cultured in the condition as previously described with either GITRL protein (1 μg/mL) or control protein for 72 hours. IL-21 production **F.** and IL-21 mRNA expression **G.** was detected by ELISA and qRT-PCR, respectively, every 24 hours.**H., I.** Naïve CD4^+^ T cells were cultured in the conditions previously described and treated with either GITRL protein (1 μg/mL) or a control protein for 72 hours in the presence or absence of Stattic (20 μM). DMSO was the solvent of Stattic. IL-21 production **H.** and IL-21 mRNA expression **I.** was detected by ELISA and qRT-PCR, respectively. Data are mean±SD. *, *P* < 0.05; ***, *P* < 0.001.

After treatment with 0.5 or 1.0 μg/mL GITRL protein, the phosphorylation of STAT3 Tyr705 and Ser727 increased in the activated CD4^+^T cells (Figure 2A). Additionally, the phosphorylation level of STAT3 Tyr705 was accelerated with GITRL protein after 20 or 40 min. The phosphorylation level of the Ser727 site was higher after a 10 or 20 min incubation with GITRL protein (Figure 2B). To explore if p38 MAPK modulates the phosphorylation of STAT3, activated CD4^+^T cells were pretreated with the p38 MAPK inhibitor and treated with GITRL for 20 min. There was decreased activity of STAT3 on Tyr705 and Ser727 with pretreatment of the p38 MAPK inhibitor (Figure 2C). These data show that GITRL protein promotes the phosphorylation of STAT3 *via* p38 MAPK.

To study the function of STAT3 during GITRL-induced Th17 cell differentiation, we observed the expansion of Th17 cells in the presence or absence of a STAT3 inhibitor. Compared with the supernatant from the developing Th17 cells induced by GITRL, IL-17 production was significantly reduced in the presence of the STAT3 inhibitor (Figure 2D). The proportion of Th17 cells was also decreased by the STAT3 inhibitor in this condition (Figure 2E). Next, we found that the concentration of IL-21 and relative expression of IL-21 mRNA reached its peak after incubating with GITRL for 72 hours (Figure 2F, 2G). Notably, the increase in IL-21 production and IL-21 mRNA expression by GITRL protein could be decreased with the STAT3 inhibitor (Figure 2H, 2I).

Therefore, it may be inferred that GITRL protein facilitated the activation of STAT3 on Tyr705 and Ser727 *via* p38 MAPK and further induce Th17 differentiation *in vitro*.

### P38 MAPK inhibitor alleviates arthritis progression in GITRL-treated CIA mice

**Figure 3:**
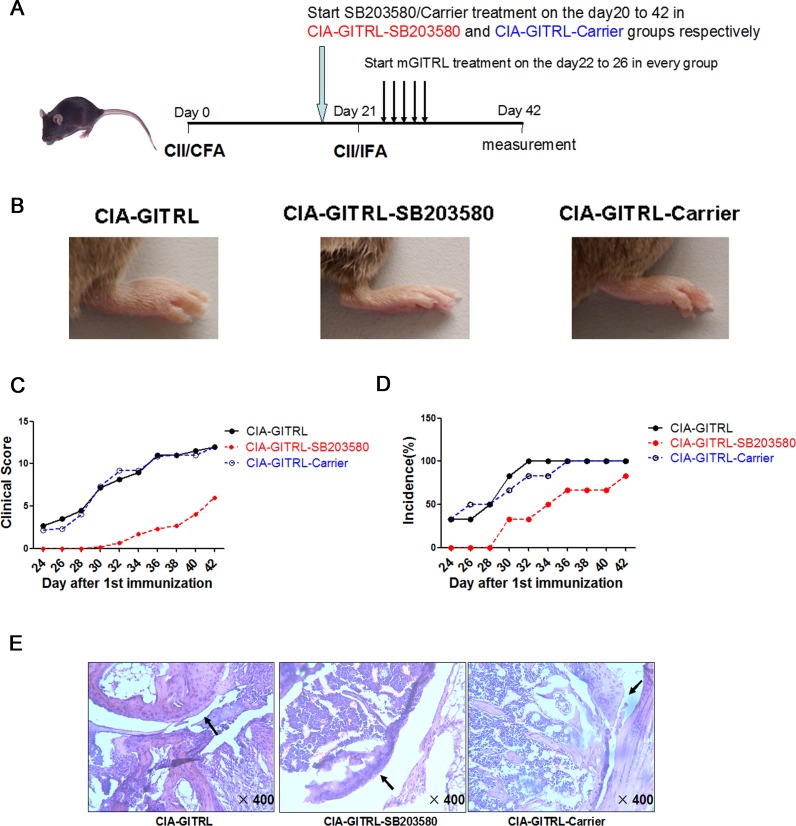
P38 MAPK inhibitor alleviates arthritis progression in the GITRL-treated CIA mice **A.** The scheme of CIA induction, GITRL protein and SB203580 administration. The DBA/1J mice were immunized with CII/CFA on day 0 and boosted with CII/IFA on day 21. The CIA-GITRL group was intravenously injected with GITRL protein (20 μg per mouse) daily for 5 consecutive days (from day 22 to day 26). In addition to being injected with GITRL protein, the CIA-GITRL-SB203580 and CIA-GITRL-Carrier group received 2.5 mg/kg/d SB203580 by ip injection in a total volume of 200 μL or an equal volume of carrier every day from the 20th day after first immunization. The mice were sacrificed on day 42 for various experiments (*n* = 6 for each group). **B.** Photographs of the hind ankle of the mice receiving the different treatments. **C.** The incidence of arthritis development of the immunized mice treated with GITRL (CIA-GITRL), GITRL and SB203580 (CIA-GITRL -SB203580), or GITRL and Carrier (CIA-GITRL-Carrier) was monitored every 2 days.**D.** The clinical scores of arthritis severity were assessed in the CIA-GITRL, CIA-GITRL -SB203580 and CIA-GITRL-Carrier groups. **E.** The inflamed joints were sectioned for hematoxylin and eosin staining. Representative sections of joint tissue from each treatment group are shown (original magnification,×400).

To define the function of p38 MAPK in GITRL-treated CIA mice, the mice were divided into groups. The scheme of CIA induction and treatment is depicted in Figure 3A. In the CIA-GITRL-SB203580 group, arthritis symptoms were alleviated by the p38 MAPK inhibitor. Changes included later onset of arthritis, reduced symptoms, and decreased clinical scores and incidence of disease (Figure 3B-3D). Moreover, histopathological analysis revealed less pronounced synovial inflammation, cartilage damage, and bone erosion in the joint tissues of mice in the CIA-GITRL-SB203580 group (Figure 3E).

**Figure 4:**
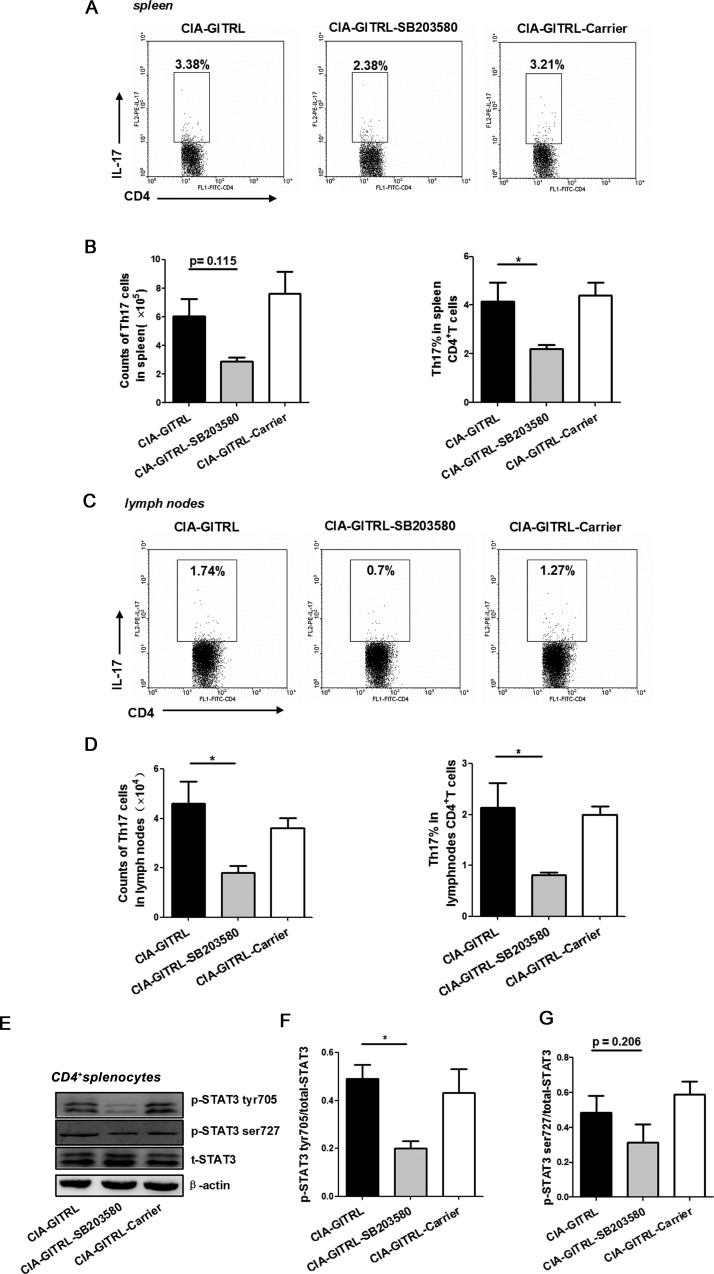
The p38 MAPK inhibitor reduced the Th17 cell response in the GITRL-treated CIA mice **A.** The frequency of Th17 cells in spleens from CIA mice with different treatments was analyzed by flow cytometry. The percentages of Th17 cells are indicated in representative dot plots.**B.** The total number (left) or percentages (right) of Th17 cells in spleens from three treatment groups are shown. **C.** The frequency of Th17 cells in draining lymph nodes from CIA mice with different treatments was analyzed by flow cytometry; percentages of Th17 cells are indicated in representative dot plots.**D.** The total number (left) and percentages (right) of Th17 cells in lymph nodes from three treatment groups were shown. **E.** The phosphorylation level of STAT3 Tyr705 and Ser727 in the spleen CD4^+^T cells of the mice from the different groups was detected by Western blot. The results indicated are in a representative photo.**F., G.** The phosphorylation level of STAT3 Tyr705 **F.** and Ser727 **G.** in the spleen CD4^+^T cells of the mice from the different groups. Data are mean±SD. *, *P* < 0.05.

Next, the number and frequency of Th17 cells were examined in the spleens and draining lymph nodes of the three groups of CIA mice. The percentage of Th17 cells in the spleens were significantly decreased in mice from the CIA-GITRL-SB203580 group compared with the CIA-GITRL group. The reduction of total number of Th17 cells in the CIA-GITRL-SB203580 group was non-significant, but it has a slight trend toward significance (*p* = 0.115) (Figure 4A, 4B). The total number and frequency of Th17 cells in the draining lymph nodes were markedly decreased in the CIA-GITRL-SB203580 group compared with the CIA-GITRL group (Figure 4C, 4D). In addition, the CD4^+^T cells from the spleens of the CIA mice were detected by the phosphorylation of STAT3 on Tyr705 and Ser727 sites with a Western blot. The CIA-GITRL-SB203580 group had a significantly lower phosphorylation level of STAT3 on the Tyr705 site in their CD4^+^T cells from the spleens, but the phosphorylation level of STAT3 on the Ser727 site has no difference (*p* = 0.206) (Figure 4E-4G). These results suggest that p38 MAPK inhibitor may inhibit arthritis progression and reduce Th17 cells in GITRL-treated CIA mice.

### The p38 MAPK phosphorylation is upregulated in CD4^+^T cells from RA patients

**Figure 5:**
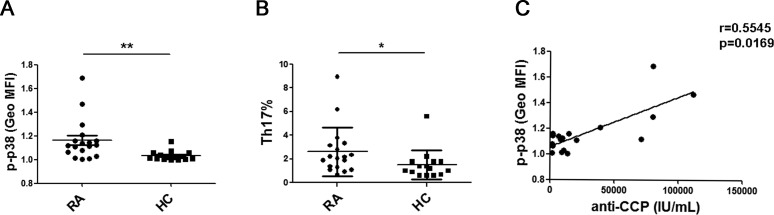
P38 MAPK phosphorylation was upregulated in the CD4^+^T cells from RA patients **A.** PBMCs from 15 healthy controls and 18 patients were stained for phospho-p38 MAPK. The mean fluorescence intensity (MFI) of phosphor-p38 MAPK in CD4^+^T cells was compared between the RA patients and the healthy controls. All the values were gated on the CD3^+^CD4^+^ T cells.**B.** PBMCs from 15 healthy controls and 18 patients were stimulated for 5 hours with PMA/ionomycin and then stained for the cell surface molecule CD3 and CD8 as well as intracellular IL-17 and analyzed by flow cytometry. Percentages of Th17 cells were compared between RA patients and healthy controls.**C.** The correlation between the MFI of phospho-p38 MAPK in CD4^+^T cells and serum level of anti-CCP antibody in RA patients (r = 0.5545, *p* = 0.0169). Each data point represents an individual subject. *, *P* < 0.05; **, *P* < 0.01.

Previously, we found that serum GITRL is significantly increased in and is positively correlated with serum IL-17A in RA patients. Therefore, we investigated the expression of the p38 MAPK in CD4^+^T cells and analyzed the percentage of Th17 cells from the peripheral blood of RA patients. Remarkably, the phosphorylation of p38 MAPK in the CD4^+^T cells and the percentage of Th17 cells from RA patients was significantly higher than the cells isolated from the healthy controls (Figure 5A, 5B). Furthermore, we analyzed the correlation between the serum level of anti-CCP antibody and phosphorylation of p38 MAPK in the CD4^+^T cells from RA patients. There was a positive correlation between these two parameters (r = 0.5545, *p* = 0.0169) (Figure 5C).

## DISCUSSION

GITR-GITRL cross-linking costimulated naïve and activated T cells and resulted in MAPK activation. The MAPK family includes three main members: p38 MAPK, extracellular signal-regulated kinase (ERK) and c-Jun N-terminal kinase (JNK). The individual MAPKs are activated by different stimuli and are implicated in developing immune responses. Studies have shown the p38 MAPK signaling pathway is essential for IL-17 production in CD4^+^T cells, and the regulation of p38 MAPK activity *in vivo* could influence the process and severity of EAE [19-21]. On the contrary, several studies have certified a negative function of ERK signaling during the generation of Th17 cells. Inhibition of ERK signaling enhanced the generation of Th17 cells *in vitro* and increased pathogenic potency *in vivo* [28]. Additionally, researchers have found that JNK plays a dual function in Th17 cell development. JNK inhibition dramatically enhanced IL-17 and CCR6 expression in developing Th17 cells, indicating that the JNK pathway controls Th17 cell differentiation. Over-expression of JNK enhanced Rorc expression in the absence of TGF-β (Th0+IL-6) in primary T cells [19]. Nevertheless, the involvement of p38 MAPK in GITRL induced Th17 cell differentiation has not been described. Our data showed that GITRL could enhance the phosphorylation of p38 in CD4^+^T cells. Meanwhile, treatment of p38 MAPK inhibitor restrains GITRL-induced Th17 cell expansion in a dose-dependent manner. Thus, p38 MAPK activity plays an important role in the regulation of Th17 cell differentiation induced by GITRL protein *in vitro*. Recently, a study has evaluate p38 MAPK deficiency in CIA model and found that p38γ/δ regulates the arthritis in CIA mice *via* altered T cell responses to CII and the level of IL-17 in mouse paws [29]. These results suggest an important role of p38 MAPK during the process of CIA. According to our results, we inferred the alteration of p38 MAPK activity may influence GITRL protein-induced arthritis *in vivo*. To define the function of p38 MAPK during GITRL protein-induced arthritis *in vivo*, a p38 MAPK inhibitor or control was administered to GITRL-treated CIA mice. Interestingly, the administration of the p38 MAPK inhibitor could prevent GITRL-treated arthritis progression, including decreasing clinical scores and incidence of CIA. Importantly, the increase of Th17 cells in GITRL-treated CIA mice was reduced by administration of the p38 MAPK inhibitor. Thus, these findings reveal a critical role for P38 MAPK in driving the development of Th17 cells in GITRL-treated CIA mice.

In patients with RA, Th17 cells and IL-17 drive both inflammation and joint damage. The elevated serum level of GITRL is positively correlated with serum concentration of IL-17 [16]. Consistent with the function of p38 MAPK activity in GITRL-induced Th17 cell differentiation *in vitro* and arthritis pathogenesis in CIA, a significantly higher phosphorylation level of p38 MAPK was detected in the peripheral CD4^+^T cells from the RA patients than in those from the healthy control subjects. There was a positive relationship between the serum level of anti-CCP antibody and the p38 MAPK phosphorylation in CD4^+^T cells from the RA patients. These results suggest that the enhancement of p38 MAPK phosphorylation and the increase in Th17 cells may be involved in RA pathogenesis. However, whether p38 MAPK phosphorylation in CD4^+^T cells could serve as a biomarker for disease activity remains to be investigated.

What is the downstream signaling of GITRL-mediated activation of p38 MAPK in Th17 cells? Recently, phosphorylation at the Ser727 site of STAT3 by all three MAPK family members has been reported in response to different extracellular stimuli, suggesting a cross-talk between MAPK cascades and JAK-STAT pathways [26, 27, 29]. Importantly, STAT3 is a key transcription factor for Th17 differentiation. Full activation of STAT3 requires dual phosphorylation of Tyr705 and Ser727 sites [23, 30, 31]. Interestingly, the phosphorylation of STAT3 in activated CD4^+^T cells at Tyr705 and Ser727 was enhanced after the treatment with GITRL protein, and the enhancement of phosphorylation of STAT3 by GITRL was reversed once treated with the p38 MAPK inhibitor. Meanwhile, Th17 cell expansion and IL-21 production induced by GITRL could be restrained by the STAT3 inhibitor. Additionally, our results showed that GITRL could reinforce the phosphorylation of STAT3 on both Tyr705 and Ser727 *via* promoting p38 MAPK activity *in vitro*. The treatment of the p38 MAPK inhibitor in the GITRL-treated CIA mice markedly decreased the phosphorylation of STAT3 on Tyr705 in CD4^+^T cells, but the decrease at the Ser727 site was small. Additionally, the role of GITRL-mediated activation of p38 MAPK in directly contributing to phosphorylation of STAT3 in Th17 cells remains to be clarified.

Taken together, these results indicate that GITRL could promote Th17 cell differentiation by p38 MAPK and STAT3 signaling in autoimmune arthritis, which is considered a central pathogenesis of autoimmune disorders.

## MATERIALS AND METHODS

### Mice

DBA/1J mice (6- to 8-week-old, male) were purchased from the Shanghai Slac Laboratory Animal Company (Shanghai, China) and maintained in a specific pathogen-free animal facility at Jiangsu University. All the animal experimental procedures used in this study were approved by the Jiangsu University Animal Ethics and Experimentation Committee.

### Patients

Eighteen patients with RA ranging from 31 to 84 years old (52.56 ± 17.32, data correspond to the arithmetic mean±SD) were included in this study. All the patients met the American College of Rheumatology (ACR) 1987 and The European League against Rheumatism (EULAR) 2009 revised criteria for the classification of RA. Fifteen healthy subjects free of rheumatic disease, chronic pain, cardiovascular complaints, or other chronic inflammatory diseases were the control. The peripheral blood was taken from patients and healthy subjects after fasting for 8 hours in the morning. The serum concentration of the anti-CCP antibody was measured using a standard ELISA and ELISA reader (Bio-Rad). Peripheral blood mononuclear cells (PBMCs) were prepared from all the patients and the healthy controls for the subsequent measurements.

All samples were taken in accordance with regulations and with the approval of the Affiliated People's Hospital of Jiangsu University.

### Cell culture

CD4^+^ naïve T cells were purified from mice spleen cell suspensions by magnetic beads using a CD4^+^CD62L^+^T Cell Isolation Kit II (Miltenyl Biotec, DE). Naïve CD4^+^T cells were activated using 1 μg/mL anti-CD3 mAb (Biolegend) and 1 μg/mL GITRL protein for 72 hours. The activated cells were washed and restimulated with GITRL protein at 37°C. The activated CD4+T cells were pretreated with 5 μM of the p38 MAPK inhibitor SB203580 (Cell Signaling Technology, CST) for 30, 60, 90, and 120 min and restimulated with GITRL protein for 20 min. Cultured cells were prepared for Western blot analysis. Recombinant GITRL protein was produced and purified as previously described [16].

For Th17 cell differentiation, naïve CD4^+^T cells were cultured in the presence of 2.5 ng/ml TGF-β (PeproTech), 30 ng/ml IL-6 (PeproTech), 30 ng/ml IL-23 (PeproTech), 5 μg/ml anti-IFN-γ (Biolegend) and 5 μg/ml anti-IL-4 (Biolegend) in a 24-well plate precoated with anti-CD3 mAb (1 μg/mL) and treated with either GITRL protein or control protein for 72 hours in the presence or absence of SB203580.

### Induction and evaluation of collagen-induced arthritis (CIA)

Briefly, 100 μL (1 mg/mL) of bovine type II collagen (CII; Chondrex) was emulsified with an equal volume of complete Freund's adjuvant (CFA, 2 mg/mL; Chondrex) and administered intradermally at the base of tail into DBA/1J mice. On day 21, a booster emulsion prepared with CII and incomplete Freund's adjuvant was intradermally administered near the primary injection site. Mice received 2.5 mg/kg/d SB203580 by IP injection in a total volume of 200 μL or an equal volume of carrier every day from the 20th day after the first immunization. Beginning on day 21, the mice were scored for arthritis severity every two days as previously described [22]. Eighteen mice were randomly and equally separated into three groups. The first group was treated with the mGITRL protein (CIA-GITRL group), the second group with mGITRL and the p38 inhibitor SB203580 (CIA-GITRL-SB203580 group), and the last group with mGITRL and the p38 inhibitor solvent (CIA-GITRL-Carrier group).

### Western blot analysis

Whole-cell lysates were prepared using 1-5×10^6^ cells in RIPA lysis buffer, separated by SDS-PAGE, and transferred to PVDF membranes. Primary Abs used for Western blot analysis included anti-phospho-p38 MAPK (CST), anti-phospho-STAT3 (Tyr705; CST), anti-phospho-STAT3 (Ser727; CST), anti-STAT3 (Santa Cruz), and anti-actin (Abcam). Anti-rabbit-HRP and anti-mouse-HRP (Jackson ImmunoResearch Laboratories) were used as secondary Abs. The bands were detected by chemiluminescent detection (Champion Chemical) according to the manufacturer's instructions.

### Reverse transcription and real-time PCR

Total RNA was extracted from splenocytes from the mice or from cultured CD4^+^T cells using TRIzol (Invitrogen). The RNA was subjected to reverse transcription, and was performed as previously described (TOYOBO) [33]. Real-time PCR was performed in duplicate using the Bio-Rad SYBR green super mix (Bio-Rad). The expression value was normalized to β-actin in the same sample. The sequences of the primer pairs are as follows: mIL-17, forward, 5′-TCCAGAAGGCCCTCAGACTA-3′, reverse-AGCATCTTCTCGACCCTGAA; mRORγt: forward, 5′-GAAAGCAGGAGCAATGGAAG-3′, reverse 5′-CTCCACACCACCGTATTTGC -3′; mIL-21: forward 5′-TCCAGAAGGCCCTCAGACTA-3′, reverse 5′-AGCATCTTCTCGACCCTGAA-3′; mβ-actin: forward 5′-TGGAATCCTGTGGCATCCATGAAAC-3′, reverse 5′-TAAAACGCAGCTCAGTAACAGTCCG-3′.

### Flow cytometry

The isolation of PBMC was performed as previously described [34]. Staining markers were identified with relevant fluorochrome-conjugated anti-mouse-CD4, anti-mouse-IL-17A, anti-human-CD3, anti-human-CD8, and anti-human-IL-17A mAb (eBioscience,) or Phospho-p38 MAPK Rabbit mAb and anti-rabbit IgG (CST). Intracellular staining for Th17 cells or phospho-p38 MAPK was performed as previously described [16]. The immunostained cells were analyzed using a FACS Calibur flow cytometer (BD Biosciences).

### ELISA

To detect IL-21 and IL-17A protein expression, ELISAs were performed with cytokine-specific kits from eBioscience according to the manufacturer's instructions.

### Histological analysis

Murine joint tissue specimens were fixed in 10% buffered formalin, followed by decalcification in 15% formic acid overnight before being embedded in paraffin. Tissue sections (4 μm thick) were prepared for hematoxylin and eosin staining.

### Statistical analysis

Statistical significance was determined by a one-way analysis of variance or Student's *t*-test. A Mann Whitney *U* test was used for the statistical analysis of human samples. Correlations between variables were determined by Spearman's correlation coefficient. *P* values < 0.05 were considered significant. Data were analyzed with GraphPad Prism5 software and SPSS17.0 software.
